# Colonic Angiolipoma: A Rare Cause of Chronic Anemia and Rectal Bleeding

**DOI:** 10.7759/cureus.56678

**Published:** 2024-03-22

**Authors:** Kawkab Alharbi, Ahmad Bilal

**Affiliations:** 1 General Surgery, Princess Noura bint abdulrahman University, Riyadh , SAU; 2 General Surgery, Al-Dar Hospital, Medina, SAU

**Keywords:** lapraocopic hemicolectomy, hematochezia, anemia, gastrointestinal neoplasms, angiolipoma

## Abstract

Angiolipomas are rare, benign tumors characterized by a mixture of adipose tissue and blood vessels, distinguishing them from lipomas. This case involves a 52-year-old woman with no significant medical history who presented with generalized weakness, fatigue, and intermittent, painless rectal bleeding over six months, initially dismissed as hemorrhoidal. Despite exhibiting mild pallor and trace rectal bleeding upon examination, significant iron-deficiency anemia was diagnosed through laboratory tests. Incorporating colonoscopy and computed tomography, the diagnostic process identified a 2 cm submucosal lesion in the ascending colon, characterized as a well-defined, fat-density mass. Histopathological analysis following surgical resection confirmed the diagnosis of a colonic angiolipoma. The patient's recovery, marked by the resolution of symptoms and normalization of hemoglobin levels, underscores the effectiveness of surgical treatment. This case highlights the diagnostic challenges posed by colonic angiolipomas due to their nonspecific symptoms. It emphasizes the importance of considering such rare entities in the differential diagnosis of gastrointestinal symptoms. This approach facilitates prompt and appropriate treatment, enriching the limited literature and advocating for clinical vigilance and interdisciplinary diagnostic strategies.

## Introduction

Angiolipoma, an encapsulated subcutaneous benign tumor, consists of adipose and proliferated blood vessels, distinguishing it histologically from a lipoma. It is classified based on the ratio between adipose and vascular tissues into predominantly lipomatous or angiomatous types [1]. While angiolipomas commonly occur on the extremities and trunk, they can also present in gastrointestinal tract organs, including the stomach, small intestine, and colon, albeit rarely. In the context of the colon, angiolipomas account for approximately 1% of all benign colonic masses [1,2].

The clinical presentation of colonic angiolipomas often includes symptoms directly correlated with the size of the masses, which may range from vague abdominal pain to altered bowel habits, nausea, vomiting, bloody stools, and anemia [1]. These symptoms highlight the variability and non-specific nature of angiolipoma presentations, complicating the diagnostic process. Diagnostic imaging techniques, such as barium enema, abdominal ultrasound, computed tomography, and magnetic resonance imaging, are crucial in determining the lesion's size and location within the gastrointestinal tract [2]. However, definitive confirmation of angiolipomas is achieved only through postoperative histological evaluation after surgical resection [2]. Given the complexity and rarity of colonic angiolipomas, including them in the differential diagnosis of gastrointestinal symptoms is essential for timely and appropriate management.

## Case presentation

We present a case involving a 52-year-old female with no significant past medical history, who came to the outpatient department complaining of generalized weakness and fatigue progressively worsening over six months. Additionally, she experienced intermittent, painless rectal bleeding during the same period, which she initially attributed to hemorrhoids, thus delaying seeking medical advice. However, the persistence and gradual exacerbation of her symptoms eventually prompted her to seek medical attention. She denied any associated weight loss, fever, or changes in bowel habits. Her family history was non-contributory, and she had no known drug allergies. She did not take any regular medications, and her surgical history was unremarkable.

Physical examination revealed a mildly pale woman in no acute distress. Her vital signs were within normal limits. The abdominal examination was unremarkable, with no palpable masses or tenderness noted. A rectal examination revealed no external hemorrhoids, fissures, or masses; however, there was a trace of bright red blood on the glove. The remainder of her physical examination, including cardiovascular, respiratory, neurological, and musculoskeletal assessments, was normal.

Given her presentation of chronic anemia and rectal bleeding, an initial workup was initiated. Laboratory investigations revealed significant anemia with a hemoglobin level of 8.2 g/dL (normal range: 12-15.5 g/dL). Her mean corpuscular volume was within normal limits, indicating a normocytic anemia. Iron studies showed iron deficiency with a low serum iron level of 30 μg/dL (normal range: 50-170 μg/dL), a low ferritin level of 15 ng/mL (normal range: 15-200 ng/mL), and a high total iron-binding capacity. The remainder of the complete blood count, coagulation profile, and biochemistry panel, including liver and renal function tests, were within normal limits (Table 1).

**Table 1:** Laboratory test results and reference ranges for the patient All values are within normal ranges except for hemoglobin, hematocrit, iron, and total iron binding capacity, which indicate iron deficiency anemia.

Laboratory Test	Units	Patient Value	Reference Range
Hemoglobin	g/dL	8.2	12.0 - 15.5
Hematocrit	%	24.5	36.0 - 46.0
Mean Corpuscular Volume	fL	82	80 - 100
White Blood Cell Count	x10^9^/L	7.2	4.0 - 10.0
Platelets	x10^9^/L	250	150 - 400
Iron	µg/dL	30	50 - 170
Total Iron Binding Capacity	µg/dL	480	250 - 450
Ferritin	ng/mL	15	15 - 200
Vitamin B12	pg/mL	500	200 - 900
Folate	ng/mL	12.0	2.0 - 20.0
Creatinine	mg/dL	0.9	0.6 - 1.2
Blood Urea Nitrogen	mg/dL	14	7 - 20
Aspartate Aminotransferase	U/L	20	10 - 40
Alanine Aminotransferase	U/L	18	7 - 56
Alkaline Phosphatase	U/L	90	40 - 130
Carcinoembryonic Antigen	ng/mL	1.2	<5.0

**Figure 1:**
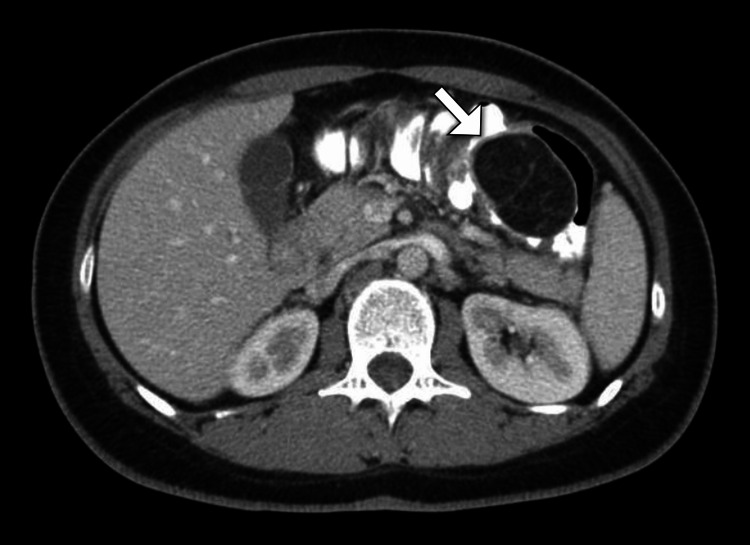
Axial CT scan of the abdomen demonstrates a lesion with fat-density characteristics (arrow) located within the ascending colon CT: computed tomography

To further investigate the source of bleeding and anemia, the patient underwent a colonoscopy, which uncovered a 2 cm submucosal lesion with a smooth surface in the ascending colon. The lesion bled easily upon contact. Biopsies were taken, and the remainder of the colonoscopy was unremarkable. An abdominal computed tomography scan with contrast revealed a well-circumscribed, fat-density mass within the wall of the ascending colon (Figure 1). There were no signs of bowel obstruction, and no other abnormalities were identified.

**Figure 2:**
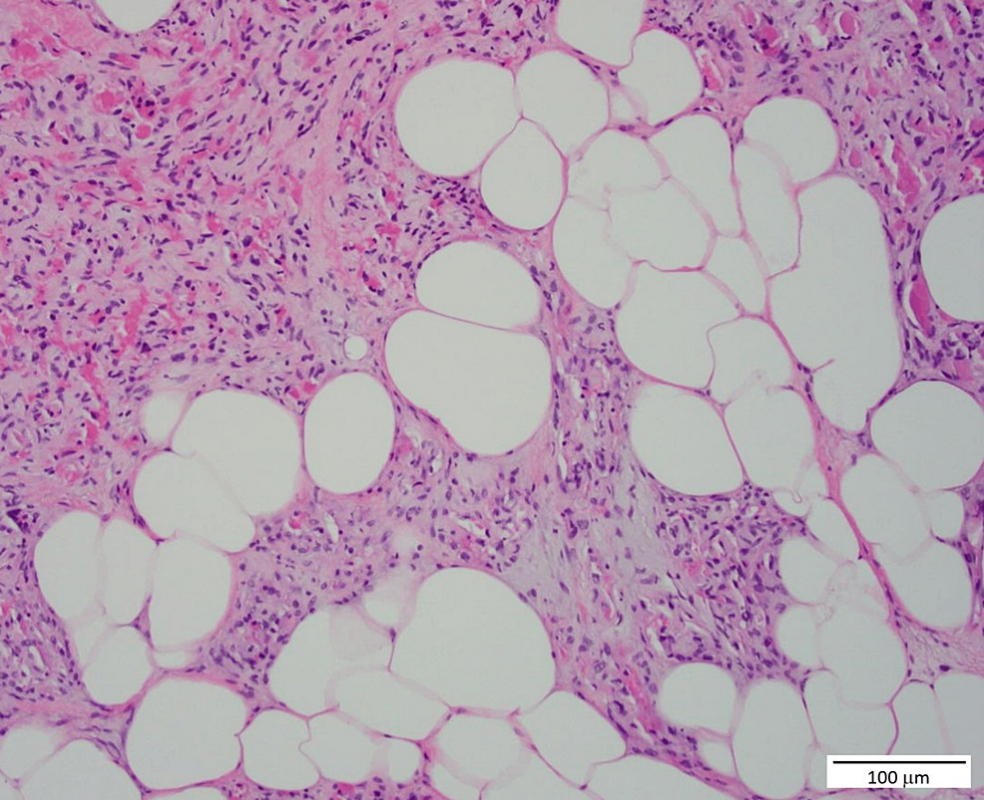
Histopathological examination shows mature adipose tissue with interspersed proliferating blood vessels (H&E stain, 20× magnification) H&E: hematoxylin and eosin staining

The differential diagnosis included other causes of gastrointestinal bleeding such as colorectal carcinoma, adenomatous polyps, diverticulosis, and inflammatory bowel disease. However, the imaging and histopathological findings were most consistent with a diagnosis of colonic angiolipoma. Considering the patient's symptomatic anemia and the risk of recurrent bleeding, surgical resection was deemed necessary. The patient underwent a laparoscopic right hemicolectomy. The postoperative course was uneventful, and she was discharged on postoperative day 5. Histopathological examination of the resected specimen confirmed the diagnosis of colonic angiolipoma, characterized by mature adipose tissue and proliferating blood vessels within the colonic wall (Figure 2).

During follow-up visits, the patient reported significant improvement in her symptoms, with resolution of the anemia confirmed by normal hemoglobin levels. She experienced no further episodes of rectal bleeding.

## Discussion

The case of a 52-year-old female presenting with chronic anemia and rectal bleeding, ultimately diagnosed with a colonic angiolipoma, elucidates several critical aspects of this rare entity's management and diagnostic challenges. Angiolipomas are encapsulated tumors consisting of both adipose tissue and proliferated blood vessels, which distinguishes them histologically from lipomas. These tumors are further classified based on the ratio of adipose to vascular tissue [1-3]. Their occurrence in the gastrointestinal tract, though uncommon, poses significant clinical importance due to the potential symptoms they can cause such as abdominal pain, altered bowel habits, and chronic anemia due to occult bleeding [2,3].

The diagnostic journey in this case highlights the indispensable role of advanced imaging techniques and the necessity for histological confirmation following surgical intervention. The use of colonoscopy and computed tomography scans was pivotal in the initial detection and characterization of the lesion, underlining their value in the diagnostic algorithm for evaluating gastrointestinal symptoms. This strategy is in line with literature recommendations, advocating for a comprehensive approach using various imaging modalities to detail the lesion's characteristics before definitive surgical intervention [2-4].

Surgical resection remains the treatment of choice for managing symptomatic colonic angiolipomas or in situations where malignancy cannot be excluded. The application of minimally invasive laparoscopic techniques, in this case, mirrors the broader shift toward reducing operative morbidity and enhancing recovery times, a trend well-supported by evidence indicating that surgical resection is associated with lower risks of complications, such as perforation and bleeding, compared to endoscopic resection [2,5].

The successful postoperative recovery and resolution of symptoms observed in this patient further support the effectiveness of surgical management in cases of colonic angiolipomas. The follow-up outcomes, which include the absence of anemia or rectal bleeding recurrence, underscore the long-term success of this treatment strategy. This case provides valuable insights into the diagnosis and management of colonic angiolipomas, enriching the limited body of literature on this rare condition.

## Conclusions

In conclusion, this case report underscores the clinical significance of angiolipomas within the gastrointestinal tract, a rare entity presenting with symptoms of chronic anemia and rectal bleeding. Through detailed investigation, including advanced imaging and definitive histological confirmation, we highlight the critical role of comprehensive diagnostic evaluation and the efficacy of surgical resection in managing colonic angiolipomas. This case adds to the sparse literature on colonic angiolipomas, emphasizing the necessity for heightened clinical awareness and an interdisciplinary approach for timely diagnosis and management. It reinforces the importance of considering rare benign tumors in the differential diagnosis of common gastrointestinal symptoms, thereby facilitating prompt, targeted intervention and ensuring optimal patient outcomes. Our report aims to enhance understanding and stimulate further research into the diagnosis, treatment, and long-term management of colonic angiolipomas, contributing to improved care for patients with this rare condition.
